# Structural and Population-Based Evaluations of TBC1D1 p.Arg125Trp

**DOI:** 10.1371/journal.pone.0063897

**Published:** 2013-05-07

**Authors:** Tom G. Richardson, Elaine C. Thomas, Richard B. Sessions, Debbie A. Lawlor, Jeremy M. Tavaré, Ian N. M. Day

**Affiliations:** 1 Bristol Genetic Epidemiology Laboratories, School of Social and Community Medicine, University of Bristol, Oakfield House, Oakfield Grove, Bristol, United Kingdom; 2 School of Biochemistry, Medical Sciences Building, University of Bristol, Bristol, United Kingdom; 3 MRC Centre for Causal Analyses in Translational Epidemiology (CAiTE), School of Social and Community Medicine, University of Bristol, Oakfield House, Oakfield Grove, Bristol, United Kingdom; Wadsworth Center, United States of America

## Abstract

Obesity is now a leading cause of preventable death in the industrialised world. Understanding its genetic influences can enhance insight into molecular pathogenesis and potential therapeutic targets. A non-synonymous polymorphism (rs35859249, p.Arg125Trp) in the N-terminal TBC1D1 phosphotyrosine-binding (PTB) domain has shown a replicated association with familial obesity in women. We investigated these findings in the Avon Longitudinal Study of Parents and Children (ALSPAC), a large European birth cohort of mothers and offspring, and by generating a predicted model of the structure of this domain. Structural prediction involved the use of three separate algorithms; Robetta, HHpred/MODELLER and I-TASSER. We used the transmission disequilibrium test (TDT) to investigate familial association in the ALSPAC study cohort (N = 2,292 mother-offspring pairs). Linear regression models were used to examine the association of genotype with mean measurements of adiposity (Body Mass Index (BMI), waist circumference and Dual-energy X-ray absorptiometry (DXA) assessed fat mass), and logistic regression was used to examine the association with odds of obesity. Modelling showed that the R125W mutation occurs in a location of the TBC1D1 PTB domain that is predicted to have a function in a putative protein:protein interaction. We did not detect an association between R125W and BMI (mean per allele difference 0.27 kg/m^2^ (95% Confidence Interval: 0.00, 0.53) P = 0.05) or obesity (odds ratio 1.01 (95% Confidence Interval: 0.77, 1.31, P = 0.96) in offspring after adjusting for multiple comparisons. Furthermore, there was no evidence to suggest that there was familial association between R125W and obesity (χ^2^ = 0.06, P = 0.80). Our analysis suggests that R125W in TBC1D1 plays a role in the binding of an effector protein, but we find no evidence that the R125W variant is related to mean BMI or odds of obesity in a general population sample.

## Introduction

Obesity has become a major cause of morbidity and mortality in the industrialised world, substantially through the impact on incidence of type 2 diabetes mellitus and coronary heart disease [1]. While environmental change has driven the increase in obesity, genetic contributions may highlight aspects of pathogenesis and novel pathways susceptible to new interventions (drug or nutritional) [2]–[4].

A non-synonymous polymorphism (rs35859249, C to T) in *TBC1D1*, referred here as the R125W variant or 125W risk allele, has been previously reported to be associated with familial obesity in women [5], [6]. R125 is conserved as an Arg or Gln in mammals and locates within the first phosphotyrosine-binding (PTB) domain of TBC1D1 [5]. Stone et al. [7] initially identified a predisposition locus at 4 p15–14, in which *TBC1D1* locates, to severe obesity (Body Mass Index (BMI)>35 kg/m^2^) in pedigrees of US Caucasian females. They analysed 435 pedigrees of European descent where on average 10 subjects in each family had a BMI≥35 kg/m^2^. They identified 10 pedigrees from this sample that had a logarithm (base 10) of odds (LOD) score≥1.0 in 4 p15–14 and found strong evidence for obesity linkage at this locus in females. However, there was no prior rationale for sex differences and no gene*sex interaction tests were presented. In a subsequent study, Stone et al. [5] reported linkage of the *TBC1D1* 125W risk allele with severe familial obesity in females only, using the same 10 aforementioned pedigrees. This familial association of the R125W variant with obesity has since been replicated in a European cohort consisting of 9,714 French individuals, through the over-transmission of the 125W allele into obese offspring [6]. Using the 97^th^ percentile as their threshold for obesity, there was a borderline significant over-transmission of 125W risk allele into obese subjects (P = 0.05). After stratifying by sex they found evidence of a familial association in females (P = 0.008) but did not present any results of a gene*sex interaction test. Neither group that have observed the associations in families of probands with extreme obesity have found replication in population samples, using a sample size of 137 unrelated females (BMI≥35 kg/m^2^) and 4,634 general population individuals [5], [6].

TBC1D1 is a Rab-GTPase Activating Protein, which links the signals generated by insulin and muscle contraction to the molecular machinery facilitating glucose uptake into muscle cells [8]–[10] where the protein is most highly expressed [11]. Expression of the R125W TBC1D1 mutant in mouse skeletal muscle impaired insulin-stimulated, but not contractile-stimulated, glucose uptake [12].

Recent genetic evidence from the Swiss Jim Lambert (SJL) mouse strain, which express a truncated form of the TBC1D1 protein, suggests that TBC1D1 may have a more direct role in whole-body energy homeostasis [13] as these mice were lean and resistant to high fat diet-induced obesity. Depletion of TBC1D1 in the C2C12 muscle cell line resulted in increased fatty acid uptake and oxidation, consistent with similar observations in glycolytic muscles of SJL mice [13]. Furthermore, sequence variation at *TBC1D1* identified it as a major quantitative trait locus in distinguishing growth characteristics between chickens bred for meat-producing or egg-laying [14] additionally supporting a role for TBC1D1 in metabolism and growth.

Given the evidence that the *TBC1D1* R125W variant is associated with familial extreme obesity but does not appear to be associated with obesity in general population cohorts [5], [6] together with the physiological relevance of TBC1D1 in regulating glucose and lipid homeostasis, we set out both to (i) model the location of R125 in the first PTB domain of TBC1D1 and (ii) undertake a further study of the familial association of the R125W variant with obesity in a general population birth cohort of 2,292 white British mother-offspring pairs.

## Results

### Homology Model of TBC1D1 PTB1 Domain

**Figure 1:**
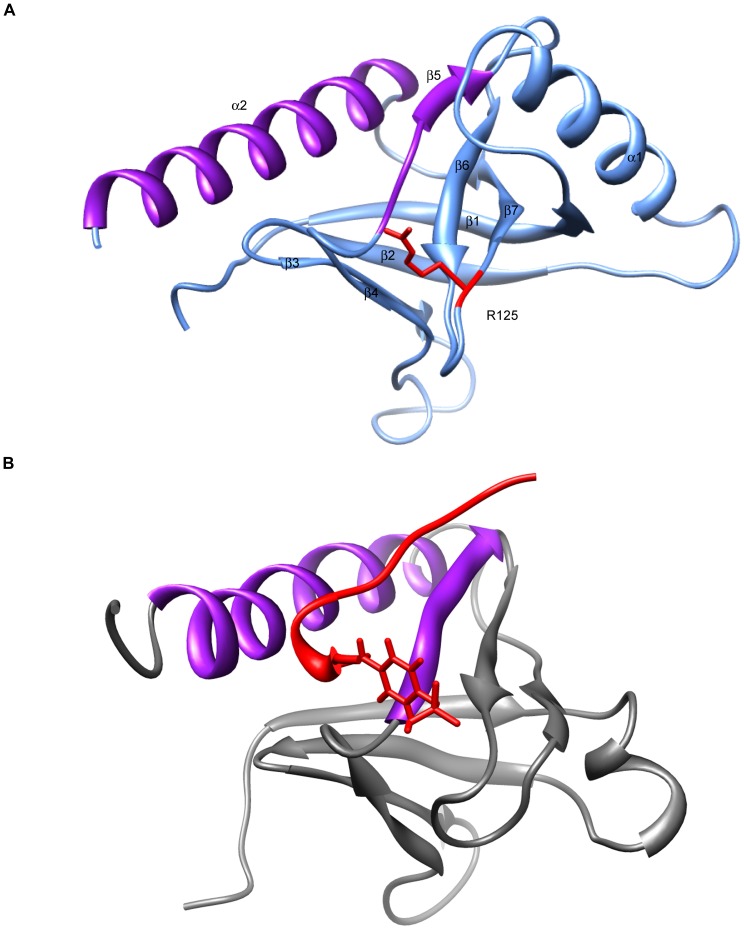
Location of R125 in the homology model of human TBC1D1 PTB1. (A) Homology based model of TBC1D1 PTB domain (residues 13–161) predicted by Robetta server (http://robetta.bakerlab.org/). The R125 residue (red) is orientated towards the cleft formed between β5 and α2 (purple). (B) Solved structure of IRS-1 PTB domain (PDB ID: 1IRS, [47]). The IL-4 phosphopeptide (red) lies within the cleft formed between the β5 and α2 (purple), a type I β turn redirects the peptide such that the phosphorylated Tyr (shown) is orientated towards the β6/β7 loop.

To gain insight into the molecular impact of an Arg to Trp substitution we generated homology models of the first PTB domain of human TBC1D1. The best available template for homology modelling TBC1D1 PTB1, the PTB domain of AIDA1 (2M38; DOI:10.2210/pdb2m38/pdb), has a sequence identity of 24% which is a little lower than the required≥30% required for reliable fold identification from a single template. However, the Hidden Markov methods applied in the homology modelling servers used here confirm that the PTB1 domain possesses the standard PTB-domain fold. For example, the HHpred E-values for the top 10 template structures are between 10^−36^ and 10^−33^ and the average of the Cα Root-mean-square deviation (RMSD) between models built with these templates and 2M38 is 2.25 Å, i.e. the same fold in each case. Despite this rather low (<25%) primary sequence homology to known PTB domain structures, models of PTB1 generated from three independent servers were very similar with only 1.2Å–2.2 Å RMSD between the C-alphas of their secondary structural elements (Table 1). The models differed more in the prediction of the loop regions (Figure S1), which is unsurprising given these regions are intrinsically more flexible and are likely to exhibit an ensemble of rapidly interconverting conformations in the physiological environment. The homology model generated by Robetta server, based on the structure of human Numb-like protein (PDB ID: 3F0W), was determined, by PROCHECK analysis, to have better geometric quality than the models created by HHpred/Modeller and I- TASSER servers (Table S1 and Method S1). The predicted model (Figure 1A) consists of the core PTB fold, a β sandwich of perpendicular antiparallel β sheets (β1–β4 and β5–β7) flanked by a C-terminal α helix (α2), with an additional α helix between β1 and β2. As shown in Figure 1A, R125 locates within a loop between β6 and β7 with the side chain capable of orienting towards the cleft formed between β5 and α2. Numerous structures solved in the presence of peptides support this groove as a canonical peptide binding site particularly for the NPX(p)Y/F motif which upon binding directs the main chain of the peptide towards the β6/β7 loop of the PTB domain [15]. Given the proximity of R125 to a putative peptide binding cleft, substitution of a positively-charged, hydrophilic arginine to a bulky, neutral, more hydrophobic tryptophan could have significant repercussions on the biological function of this domain.

**Table 1:** Comparison of homology models generated by Robetta, HHpred/MODELLER and I-TASSER servers.

Model	RSMD	
	All C alphas	C alphas of secondary structure*
MODELLER – Robetta	3.8 Å	2.0 Å
MODELLER – I-TASSER	3.4 Å	1.2 Å
I-TASSER – Robetta	4.3 Å	2.2 Å

* Manually determined C alphas of secondary structure correspond to human TBC1D1 residues 18–56, 61–79, 92–120, 128–136 and 142–162.

Before examining the associations of R125W with phenotypes in our cohort, we used a range of prediction tools that were recently evaluated [16] to assess the pathogenicity of the variant. The conclusions differed, as the Polyphen and SIFT tools predicted the variant to be harmful, whereas MutPred and SNPsGO reported it to have a neutral effect (Table 2).

**Table 2:** Evaluation of the predicted pathogenicity of R125W according to a range of prediction tools.

Prediction Tool	Effect
SIFT	DELETERIOUS
PolyPhen	DAMAGING
PolyPhen2 HumDiv	DAMAGING
PolyPhen2 HumVar	DAMAGING
PANTHER	UNKNOWN
MutPred	NEUTRAL
SNPsGO	NEUTRAL

### Study Sample Characteristics

8,228 mothers and 9,193 offspring were successfully genotyped for the R125W variant with a minor allele frequency (MAF) of 9.0% and 8.9% respectively for the 125W allele. The genotype frequencies for both mothers and children were consistent with Hardy-Weinberg equilibrium (P = 0.24 and 0.18 respectively). These MAF in mothers and offspring in our cohort were consistent with those found in previous general population studies [5], [6].

Table 3 shows the study characteristics. All offspring measurements were taken during the 15+year focus clinic (mean age: 15.5 years; range: 14.3–17.6 years). The adiposity phenotypes measured were BMI (mean: 21.4 kg/m^2^, range 14.1 kg/m^2^–39.7 kg/m^2^), waist circumference (mean: 76.5 cm, range 54.3 cm–125.9 cm) and (Dual-energy X-ray absorptiometry) DXA-assessed fat mass 14.1 kg (0.6 kg–70.9 kg). Adiposity measurements differed somewhat between males and females but the magnitude of these differences was small, despite the low p-values, which reflected the large sample size for these gender comparisons. For the mothers, BMI was the only adiposity measurement available; this was calculated from their self-report of prepregnancy height and weight given at mean age 27.7 when they were recruited in early pregnancy (mean BMI = 23.0 kg/m^2^, range = 12.5 kg/m^2^–51.6 kg/m^2^).

**Table 3:** Study Characteristics in the ALSPAC cohort.

Characteristic	Males		Females		P (no sex difference)
	N	Mean (SD)	N	Mean (SD)	
Age (years)	2,026	15.5 (0.3)	2,195	15.6 (0.3)	0.03
BMI (kg/m^2^)	2,007	21.0 (3.3)	2,158	21.7 (3.6)	<0.001
Waist Circumference (cm)	1,598	76.8 (8.6)	1,823	76.2 (8.7)	0.05
DXA Fat Mass (kg)	2,003	10.0 (6.6)	2,150	17.9 (7.6)	<0.001
Maternal Age (years)	3,421	29.0 (4.8)	3,263	28.8 (4.7)	0.04
Maternal Pre-pregnancy BMI (kg/m^2^)	3,421	23.0 (3.8)	3,263	22.9 (3.9)	0.29

Data are presented as mean (standard deviation), BMI – Body Mass Index; DXA - Dual-energy X-ray absorptiometry; BP – Blood Pressure.

### Association between R125W and Adiposity Phenotypes in ALSPAC

**Figure 2:**
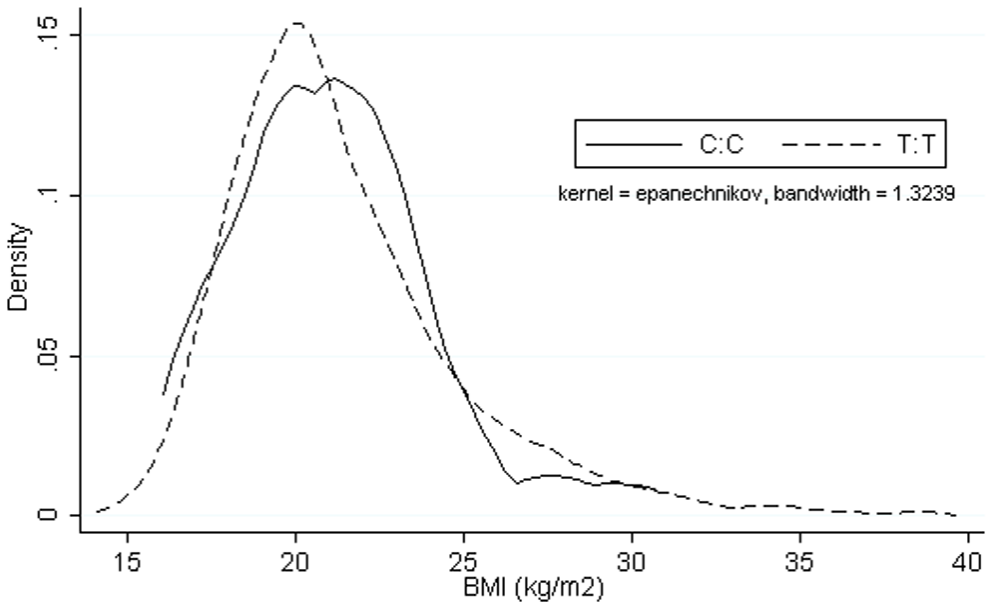
Kernel Density Plot to Show BMI Distribution at mean age 15.5 years between Homozygous Genotypes.

We found no evidence for an association of the R125W variant with mean BMI at mean age 15.5 years in the ALSPAC offspring (Table 4). The results of the Kolmogorov-Smirnov test for equality of distributions suggested there was no difference between the BMI distributions of the two homozygous genotypes (D = 0.128, P = 0.813) (Figure 2). After adjustment of multiple testing there was no strong statistical evidence that R125W was associated with any of the phenotypes that we examined as continuous traits. We also found no evidence of an association of R125W with odds of obesity at age 15.5 years, or that associations differed by sex for any phenotypes (Table S2 shows sex specific analyses; P for R125W*sex interaction all> = 0.33). Using the pre-pregnancy data from 6,684 mothers in the cohort, we found no strong evidence of association between R125W and mean BMI or odds of obesity (Table 5).

**Table 4:** Association of *TBC1D1* R125W genotypes with adiposity phenotypes in the ALSPAC Cohort.

		No. of Obs	C/C	C/T	T/T	OR/P_add_ **
	N (%)	9,193	7,600 (82.67%)	1,529 (16.63%)	64 (0.7%)	
	Sex (Male/Female)	9,193	3,949/3,651	800/729	28/36	
	BMI (kg/m^2^)	4,165	21.4 (3.5)	21.1 (3.0)	21.1 (2.8)	0.05
Continuous traits	Waist Circumference (cm)	3,421	76.7 (8.8)	75.8 (7.8)	74.3 (6.1)	0.02
	DXA Fat Mass (kg)	4,153	14.2 (8.4)	13.6 (7.2)	13.7 (7.4)	0.42
Odds of Obesity	Lean (BMI<25 kg/m^2^)*	3,404	2,793	588	23	reference
	Obesity (BMI≥30 kg/m^2^)	155	137	17	1	0.63 (0.39–1.01), P = 0.06

Data are presented as mean (standard deviation), BMI – Body Mass Index; DXA - Dual-energy X-ray absorptiometry; BP – Blood Pressure.

* Odd Ratios are calculated in comparison to lean subjects based on the additive model of inheritance. Lean, overweight and obesity cut-offs points are based on IOTF equivalents for offspring aged 15.5.

** **The Bonferroni corrected p-value equivalent to 0.05 is 0.0125.**

**Table 5:** *TBC1D1* R125W Genotype Frequencies of Mothers in the ALSPAC Cohort according to Overweight and Obese status.

	BMI Category	No. of Obs	C/C	C/T	T/T	OR/P_add_ **
N (%)	-	6,684	5,540 (82.88%)	1,099 (16.44%)	45 (0.68%)	
Association across Genotypes	BMI (kg/m^2^)	6,684	23.0 (3.8)	22.8 (3.9)	23.1 (3.9)	0.19
Odds of Obesity	Lean (BMI<25 kg/m^2^)*	5,261	4,353 (82.74%)	873 (16.59%)	35 (0.67%)	reference
	Obesity (BMI≥30 kg/m^2^)	366	301 (82.24%)	64 (17.49%)	1 (0.27%)	1.01 (0.77–1.31), P = 0.96

Data are presented as mean (standard deviation), BMI – Body Mass Index.

* Odd Ratios are calculated in comparison to lean subjects based on the additive model of inheritance.

** **The Bonferroni corrected p-value equivalent to 0.05 is 0.025.**

### Familial Association between R125W and Obesity

To investigate the over-transmission of the R125W risk allele from heterozygous mothers to affected (i.e. obese) offspring or from affected mothers to heterozygous offspring we applied the Transmission Disequilibrium Test (TDT). We found no evidence of an association of R125W with familial obesity for any of the different thresholds used to define obesity (Table 6).

**Table 6:** Results of Transmission Disequilibrium Test Assessing Association between R125W Variant and Familial Obesity.

Affected Case	BMI Percentile Threshold for Obesity	McNemar's χ^2^ Statistic	P-value	McNemar's χ^2^ Statistic (Females)	P-value	McNemar's χ^2^ Statistic (Males)	P-value
Offspring	IOTF	0.06	0.80	0.00	1.00	0.14	0.71
	97^th^	0.67	0.41	2.00	0.16	0.00	1.00
	90^th^	2.29	0.13	1.67	0.20	0.69	0.41
	75^th^	0.05	0.83	0.349	0.56	0.95	0.33
	50^th^	0.26	0.61	0.24	0.62	0.05	0.83
Mothers	IOTF	0.05	0.83	0.40	0.53	0.82	0.37
	97^th^	0.00	1.00	0.14	0.71	0.20	0.66
	90^th^	0.47	0.49	0.00	1.00	0.80	0.37
	75^th^	1.09	0.30	1.20	0.27	0.18	0.67
	50^th^	1.03	0.31	0.11	0.75	1.15	0.28

## Discussion

Our homology modelling locates R125 within a region of a PTB domain predicted to mediate protein-protein interactions; as such a tryptophan substitution would be anticipated to have deleterious functional consequences. We found no strong evidence that the *TBC1D1* R125W variant was associated with adiposity phenotypes in a general population. Furthermore, we did not find any evidence for association with familial obesity.

The R125W variant of TBC1D1 lies within the first of the two PTB domains of the protein. No structure of the TBC1D1 PTB domain is currently available to provide clues as to the role of R125 in the function of the protein. However, there are several structures of other PTB domains with sufficient amino acid sequence homology to allow homology modelling of this region of TBC1D1. We employed three different modelling algorithms to obtain a predicted structure. All methods used (i) highlighted R125 as being located in a loop formed between two β-sheets within the PTB domain structure (β6 and β7; Figure 1A) and (ii) oriented the positively charged guanidinium side-chain of R125 towards the base of a groove formed between an α-helix (α2) and a β-sheet (β5). This groove is highly reminiscent of the phosphopeptide binding groove found in the Shc and Insulin Receptor-1 (IRS1) PTB domains, where the phosphotyrosine on the peptide interacts with the guanidinium side-chains of arginine residues found at the base (compare Figure 1A with the structure of the IRS1 PTB domain in Figure 1B).

Our modelling suggests that R125 in TBC1D1 plays a role in the binding of an effector protein, possibly via a mechanism that involves phosphorylation although it should be noted that several PTB domains can bind peptide ligands in a phosphorylation-independent manner [17]. In most mammals this residue is conserved as an arginine, which is positively charged, however in some (including the rat) it exists as a glutamine (Q) that is polar but uncharged. The consequences of an R125Q substitution on the binding of a peptide ligand are difficult to predict and could be subtle, however substitution of either amino acid for a tryptophan, which is a hydrophobic aromatic residue, would be expected to be highly deleterious to function either by replacing an important electrostatic interaction by a hydrophobic one, or by significantly altering the conformational preference of this loop region.

No peptides or proteins have yet been identified that bind to the N-terminal TBC1D1 PTB domain, though it has been suggested that the PTB domains of the related protein, AS160 (TBC1D4), could be involved in dimerisation of the protein [18]. These possibilities, and the consequences of the substitutions, require further experimental investigation in the case of TBC1D1. Our finding of no association between R125W and BMI, other markers of adiposity or odds of obesity, is consistent with two previous general population studies [5], [6]. The reason for the lack of association in general populations when associations have been found with familial obesity (though not in our study) is unclear. The association between the 125W allele and familial obesity was originally identified using linkage analysis amongst pedigrees primarily consisting of families from Utah [7]. The R125W variant was enriched in the individuals in 10 of these families (P = 0.000007) when compared with 846 population controls. Ethnicity and ancestry are unlikely to explain the difference between family studies and results from general population cohorts as the latter are conducted in European origin individuals and the Utah cohort has an ancestry similar with that of Europeans [19].

Another possible explanation for the difference is that we did not preselect our case and control cohorts. Stone et al. [5] selected their case families from either the Health Family Tree Program [20] or families consisting of patients who had previously undergone a gastric bypass [21]. They manually selected pedigrees for their linkage analysis that had at least two affected sisters (BMI≥35 kg/m^2^) and also carried R125W, while the control subjects were unrelated and taken from different populations [22]. Meyre et al. [6] selected their pedigrees based on at least one person having a BMI>35 kg/m^2^ and a first degree relative with BMI>30 kg/m^2^ (435 pedigrees) or pedigrees having at least one obese child (674 pedigrees). In contrast, our study was population based and included all mothers and offspring for whom we had genotype and phenotype data and did not have any inclusion or exclusion criteria based on BMI. As such, our results cannot be interpreted as evidence against the previous findings concerning TBC1D1 [5], [6].

Moreover, if the R125W effect is restricted to, or conditional on, very high BMI, then we would not have had the statistical power to detect it. Initially we only had power of 21.21% using the International Obesity Task Force (IOTF) cut off points analysing the transmission of the 125W risk allele from mothers to affected offspring. However, we were able to control/minimise this affect in our subsequent analyses by using different thresholds for obesity to increase the number of study participants being analysed. In doing so, we achieved power of 41.31% using an obesity cut off of 90%. We also analysed the transmission of the 125W risk allele from affected mothers to offspring to more extensively evaluate the hypothesis.

A further limitation of our study is that the maternal pre-pregnancy BMI was self-reported and therefore is susceptible to recall error. Since the women will not have been aware of their genotype this would be non-differential for the association examined here and would therefore have the statistical expectation of biasing results towards the null. However, the consistent null associations in offspring across a range of directly assessed adiposity measurements could not be explained by this recall error.

## Conclusion

Our protein modelling studies support a plausible role of this amino acid in the function of the N-terminal TBC1D1 PTB domain. One initial report and one replication study have suggested that R125W is associated with familial obesity in females, though biologically it remains obscure why there should be a sex difference. Our analyses find no general population effect, nor trend in our smaller number of high BMI families. Biochemical studies of the mutant protein and further studies of families exhibiting extreme obesity may yield further insight.

## Materials and Methods

### Homology Modelling

Secondary structure prediction of human TBC1D1 (GenBank accession number NP_055988.2) with Jpred3 (www.compbio.dundee.ac.uk/www-jpred/) [23], together with domain coordinates from pfam (http://pfam.sanger.ac.uk/) [24], identified residues 13–161 to contain secondary structure elements consistent with a PTB domain topology. Tertiary structure of this region was predicted by Robetta (http://robetta.bakerlab.org/) [25], HHpred/MODELLER (http://toolkit.tuebingen.mpg.de/modeller) [26], [27] and I-TASSER servers (http://zhanglab.ccmb.med.umich.edu/I-TASSER/) [28], [29] using default parameters. Root-mean-square deviation (RMSD) values were calculated from superimposition of modelled structures with InsightII (Accelrys, San Diego, CA). Geometric quality of the models was assessed, based on the Ramachandran plot of residues and main and side chain parameters, using PROCHECK [30]. USCF Chimera (version 1.7; [31]) was used for the structure visualisation.

### Cohort Description

The Avon Longitudinal Study of Parents and Children (ALSPAC) is a population-based cohort study investigating genetic and environmental factors that affect the health and development of children. The study methods are described in detail elsewhere [32], [33] (http://www.bristol.ac.uk/alspac). Briefly, 14,541 pregnant women residents in the former region of Avon, UK, with an expected delivery date between 1^st^ April 1991 and 31^st^ December 1992, were eligible to take part in ALSPAC. There were 14,062 live born children, 13,988 of whom were alive at 1 year.

Ethical approval was obtained from the ALSPAC Law and Ethics Committee and the Southmead, Frenchay, United Bristol Healthcare NHS Trust (UBHT) and Weston Research Ethics Committees. Written informed consent was obtained from parents for all measurements made. In total, genotyping was attempted on DNA from 9,020 mothers and 10,920 offspring. All genotyping was performed by KBioscience (Herts, UK).

### Data Collection

All measurements were taken by a trained research team during the 15+year focus clinic (mean age: 15.5 years; range: 14.3–17.6 years). Height was measured to the nearest 0.1 cm using a Leicester Height Measure (Holtain Crosswell, Dyfed) and weight was measured to the nearest 0.1 kg using Tanita electronic scales. Waist circumference was measured to the nearest 1 mm at the mid-point between the lower ribs and the pelvic bone with a flexible tape. DXA determined fat mass was assessed at the clinic using a Lunar Prodigy scanner (Madison, WI, USA) with pediatric scanning software.

At the time of pregnancy ALSPAC mothers were asked in a mailed questionnaire to retrospectively report their pre-pregnancy weight and their height; these were used to calculate maternal pre-pregnancy BMI.

### Genotyping

Genotyping of the *TBC1D1* variant R125W was undertaken in 9,020 mothers and 10,920 offspring. All genotyping was performed by KBiosciences (Herts, UK) using their own system of fluorescence based competitive allele-specific PCR (KASPar). Details of assay design are available from the KBiosciences website (http://www.kbioscience.co.uk).

9,193 offspring were successfully genotyped for the R125W variant. Study participants with a reported (maternal) non-white ethnic origin (N = 218) were excluded from analyses. A further 72 offspring were removed from analyses as they were either the second born child or born in a multiple pregnancy. Of the remaining 8,903 offspring, 4,165 had been successfully measured for height and weight at the 15+year focus clinic. 3,421 had their waist circumference measurement taken and 4,153 had a DXA assessed fat mass measurement recorded.

8,228 mothers were successfully genotyped for the R125W variant and 10,569 mothers with a reported white ethnic origin had filled out a questionnaire concerning their pre-pregnancy BMI. There was an overlap of 6,748 mothers who were included in both of these groups. A further 64 subjects were removed before any analysis took place due to missing data of their offspring at the pregnancy, leaving our sample size at 6,684.

From the 6,684 mothers that were included in our BMI analysis, 2,292 of them had an offspring amongst the 4,165 subjects taken from the offspring analysis. This meant we had 2,292 mother-offspring pairs available to investigate for familial association.

### Statistical Analysis

We used Pearson's χ^2^-test amongst all offspring and mothers separately to examine if the genotype distributions were consistent with Hardy-Weinberg equilibrium [34]. Direct associations between the R125W variant and obesity-related phenotypes in the offspring were assessed by linear regression using an additive model of inheritance as this was previously described as the most suitable for R125W [5]. Logistic regression was used to examine the association of R125W with odds of obesity, again using an additive model of inheritance. Multiple comparisons were adjusted for using the Bonferroni correction [35], with a p-value threshold of 0.0125 being the equivalent of the conventional 0.05. The Kolmogorov-Smirnov test for equality of distributions [36] was used to compare the BMI distributions between the two homozygous genotypes. We used the maternal data to assess the association between the R125W variant and adult (pre-pregnancy) BMI and overweight/obesity using linear and logistic models, respectively. Any possible interaction between genotype and sex in the offspring analyses was assessed using the likelihood ratio test to compare two regression models, one which was simply adjusted for sex and another which also included an interaction term for genotype*sex. Furthermore, we analysed males and females separately to verify whether associations appeared similar in males and females.

We applied the transmission disequilibrium test (TDT) [37] to investigate familial association between the R125W polymorphism and obesity. Initially, we used the International Obesity Task Force (IOTF) cut off points for obesity [38] (BMI = 28.6 kg/m^2^ in females and 29.29 kg/m^2^ in males at age 15.5) and analysed transmissions between mother and affected child. We repeated this analysis looking at mother-son and mother-daughter pairs separately to accommodate for the previous reports associating the R125W variant with familial obesity in females. It has been suggested that the TDT suffers from a lower statistical power [39], [40] compared with case-control studies of unrelated individuals; however, in our subsequent analysis we used different thresholds for obesity to increase the number of study participants being analysed and control/minimise this affect. We extended this work by classifying the mothers as the affected case and then repeated our analysis, initially using BMI cut off point of 30 kg/m^2^.

Stata 12.0 software (StataCorp, College Station, TX) was used for general statistical analyses.

### Handling of Heterozygote Transmissions

There is literature concerning the issue of missing parental data when testing for family based association [41]–[43]. However, techniques involved in these circumstances may incorporate bias into the study [44]. When using the TDT, including with genotype data for both parents, there may be ambiguity concerning which alleles are transmitted from parents who are heterozygous to offspring who are also heterozygous [45], [46]. Since paternal genotype data was not available, we used an approach based on conditional probability, which takes into account the MAF of the R125W variant, to calculate the probability that the risk allele has been transmitted to the affected offspring. The approach is detailed in the supplementary material (Method S2).

## Supporting Information

Figure S1 **Superimposed homology models of human TBC1D1 PTB1.** Superimposed homology models of TBC1D1 PTB1 (residues 13–161) from Robetta (blue), HHpred/MODELLER (green) and I-TASSER (orange) servers with the R125 side chain displayed as spheres. Shown in yellow is the PTB domain of AIDA1 (2M38; DOI:10.2210/pdb2m38/pdb) which has the highest (24%) amino acid sequence identity to the TBC1D1 PTB1 domain. UCSF Chimera (version 1.7) was used to coordinate superimposition of structures utilising the default parameters of the matchmaker function.
(TIF)

Table S1 **Ramachandran Plot analysis, define d by PROCHECK, of the homology models generated by Robetta, HHpred/MODELLER and I-TASSER servers.**
(DOCX)

Table S2 **Average Phenotype measurements according to** ***TBC1D1*** **R125W genotypes stratified by Gender in Offspring in ALSPAC cohort.**
(DOCX)

Method S1 **Assessing the stereochemical quality of the individual homology models.**
(DOCX)

Method S2 **Conditional probability for Heterozygotes.**
(DOCX)
