# Supplementary figures and images for: Structural and Population-Based Evaluations of TBC1D1 p.Arg125Trp
Source: PLoS One. 2013 May 7;8(5):e63897. doi: 10.1371/journal.pone.0063897 (PMC3646766; doi:10.1371/journal.pone.0063897)

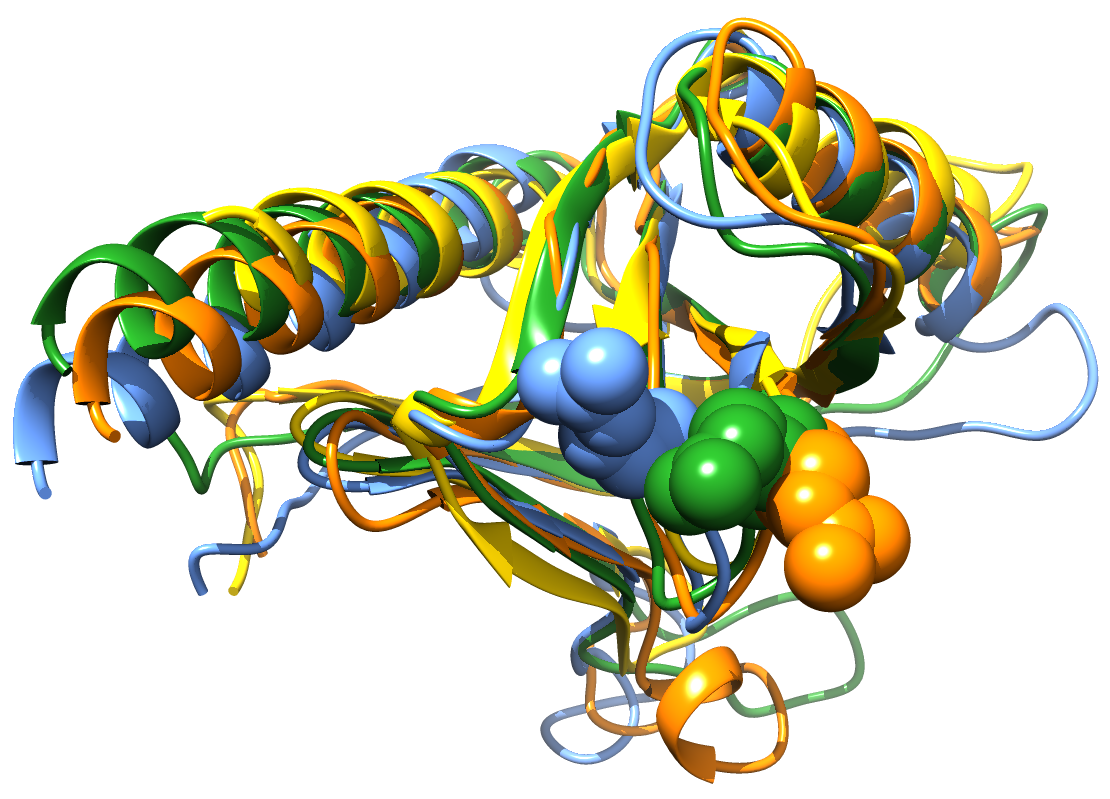

Supplement: Figure S1 — Superimposed homology models of human TBC1D1 PTB1. Superimposed homology models of TBC1D1 PTB1 (residues 13–161) from Robetta (blue), HHpred/MODELLER (green) and I-TASSER (orange) servers with the R125 side chain displayed as spheres. Shown in yellow is the PTB domain of AIDA1 (2M38; DOI:10.2210/pdb2m38/pdb) which has the highest (24%) amino acid sequence identity to the TBC1D1 PTB1 domain. UCSF Chimera (version 1.7) was used to coordinate superimposition of structures utilising the default parameters of the matchmaker function. (TIF) [file pone.0063897.s001.tif]
