# Supplementary material for: Structural and Population-Based Evaluations of TBC1D1 p.Arg125Trp
Source: PLoS One. 2013 May 7;8(5):e63897. doi: 10.1371/journal.pone.0063897 (PMC3646766; doi:10.1371/journal.pone.0063897)
Supplement: Table S1 — Ramachandran Plot analysis, define d by PROCHECK, of the homology models generated by Robetta, HHpred/MODELLER and I-TASSER servers. (DOCX) [file pone.0063897.s002.docx]

**Supplementary Table 1: Ramachandran Plot analysis, defined by PROCHECK, of the homology models generated by Robetta, HHpred/MODELLER and I-TASSER servers.**

| **Model** | **Ramachandran Plot (%** residue backbone torsion angles) | | | |
| --- | --- | --- | --- | --- |
|  | Most favoured regions | Additional allowed regions | Generously allowed regions | Disallowed regions |
| HHpred/MODELLER | 90 % | 7 % | 2 % | 1 % |
| Robetta | 86 % | 13 % | 1 % | 0 |
| I-TASSER | 84 % | 12 % | 1 % | 3 % |
