# Supplementary material for: Structural and Population-Based Evaluations of TBC1D1 p.Arg125Trp
Source: PLoS One. 2013 May 7;8(5):e63897. doi: 10.1371/journal.pone.0063897 (PMC3646766; doi:10.1371/journal.pone.0063897)
Supplement: Table S2 — Average Phenotype measurements according to TBC1D1 R125W genotypes stratified by Gender in Offspring in ALSPAC cohort. (DOCX) [file pone.0063897.s003.docx]

**Supplementary Table 2: Average Phenotype measurements according to *TBC1D1* R125W genotypes stratified by Gender in Offspring in ALSPAC cohort**

| Name of phenotype | Number of females included | Number of males included | Per allele mean difference (95% CI) in females | Per allele mean difference (95% CI) in males | P-value for interaction with sex |
| --- | --- | --- | --- | --- | --- |
| BMI (kg/m^2^) | 2,158 | 2,007 | -0.41 (-0.78 – 0.03) | -0.12 (-0.49 – 0.24) | 0.33 |
| Waist Circumference (cm) | 1,823 | 1,598 | -1.07 (-2.06 - -0.08) | -0.78 (-1.85 – 0.28) | 0.69 |
| DXA Fat Mass (kg) | 2,150 | 2,003 | -0.84 (-1.65 - -0.04) | -0.31 (-1.05 – 0.43) | 0.95 |

Data are presented as mean (standard deviation), BMI – Body Mass Index ; DXA - Dual-energy X-ray absorptiometry ;

**The Bonferroni corrected p-value equivalent to 0.05 is 0.0167**
