# Supplementary material for: Structural and Population-Based Evaluations of TBC1D1 p.Arg125Trp
Source: PLoS One. 2013 May 7;8(5):e63897. doi: 10.1371/journal.pone.0063897 (PMC3646766; doi:10.1371/journal.pone.0063897)
Supplement: Method S1 — Assessing the stereochemical quality of the individual homology models. (DOCX) [file pone.0063897.s004.docx]

**Supplementary Information 1.**

To assess the stereochemical quality of the individual homology models, generated by each of the prediction servers, the overall and residue-by-residue geometry were assessed using PROCHECK. All three models displayed main-chain and side-chain geometric parameters consistent with a 1.5 Å structure. Whilst the HHpred/MODELLER homology model showed a slightly higher percentage of backbone torsion angles in the most favoured region of the Ramachandran Plot than that produced by the Robetta server, the Robetta model did not have any residues in disallowed regions (Table S1). Based on these primary parameters we considered the homology model generated by the Robetta server to be the better quality model.
