# Supplementary material for: Structural and Population-Based Evaluations of TBC1D1 p.Arg125Trp
Source: PLoS One. 2013 May 7;8(5):e63897. doi: 10.1371/journal.pone.0063897 (PMC3646766; doi:10.1371/journal.pone.0063897)
Supplement: Method S2 — Conditional probability for Heterozygotes. (DOCX) [file pone.0063897.s005.docx]

**Supplementary Information 2.**

**Conditional probability for Heterozygotes**

Below are all potential scenarios that we needed to consider when calculating the probability that a mother with heterozygote genotype has transmitted the T allele to their offspring who also has a heterozygote genotype.

CT_M_ + CC_F_ -> CT_M_ ($\frac{1}{2}$)

CT_M_ + CT_F_ -> CT_M_ ($\frac{1}{4}$)

CT_M_ + CT_F_ -> CT_F_ ($\frac{1}{4}$)

Where M & F denote the T alleles of the mother and father respectively and the fractions represent the probability of each scenario occurring. Using the allele frequencies found in our sample, the probability of a father having genotype CC was input into the calculation as 0.8307, whereas the probability of him having genotype CT was 0.1635. We used this information to calculate the conditional probability that, given a mother’s genotype is CT, the allele transmitted to her heterozygote offspring is the T allele.

= $\frac{\left( 0.8307 \times0.5 \right)+(0.1635 \times0.25)}{\left( 0.8307 \times0.5 \right)+\left( 0.1635 \times0.25 \right)+ (0.1635 \times0.25)}$

= $\frac{0.456225}{0.4971}$ = 0.9177... = 0.918 (approx)

We used this result to help address the issue of ambiguity concerning the transmission of the risk allele from heterozygote mothers to heterozygote offspring.
